# Virtual Clinical Shadowing for Pre-Clinical Medical Students in an Emergency Medicine-Based Leadership Course

**DOI:** 10.1089/tmr.2021.0019

**Published:** 2021-10-27

**Authors:** Robert Tanouye, Jodie Nghiem, Kaela Cohan, Jane Torres-Lavoro, Kaitlin Schullstrom, Mary Mulcare, Rahul Sharma

**Affiliations:** Department of Emergency Medicine, New York-Presbyterian Hospital/Weill Cornell Medicine, New York City, New York, USA.

**Keywords:** telemedicine, medical students, pre-clinical, emergency medicine, emergency department, zoom

## Abstract

***Purpose:*** The COVID-19 pandemic limited pre-clinical medical students from participating in traditional clinical in-person shadowing. Rather than eliminating clinical shadowing from an established leadership course, we describe the experience of six pre-clinical medical students shadowing physician preceptors remotely through virtual platforms.

***Methods:*** Six pre-clinical medical students enrolled in 2020's Weill Cornell Medicine's Healthcare Leadership and Management Scholars Program were prepared with training materials for on-camera patient care. Students shadowed emergency medicine (EM) physicians providing clinical care in one of our New York Presbyterian emergency departments (EDs) and through telemedicine. Pre- and postsurveys were provided to these students.

***Results:*** From three different U.S. time zones, students were safely able to shadow EM physicians. The educational fidelity was maintained in physician–student relationships, but revealed opportunities for improvement in students' clinical learning, in ED clinical care, and in telemedicine visits.

***Conclusions:*** Virtual clinical shadowing is a viable option for pre-clinical students, when in-person options are not available. With logistical adjustments, this medium may be a long-term educational option especially for telemedicine.

## Introduction

Even before the COVID-19 pandemic, telemedicine adoption had been rising around the world.^1,2^ Paralleling this rise of telemedicine is the growth of tele-education.^3^ Before widespread use of smartphones and tablet computers, tele-education was primarily used to deliver distance learning to rural health care professionals.^4^ Leveraging such mobile devices and broadband networks, tele-education networks have been established both domestically and abroad, such as Project Extension for Community Healthcare Outcomes in the United States and Réseau en Afrique francophone pour la télémédecine in Sub-Saharan Africa, respectively.^3,5^

Although more needs to be done to validate its efficacy, initial assessments have proven favorable and tele-education has great potential as a remote learning tool.^6^ However, tele-education in the clinical arena has not yet been widely explored as a tool for U.S. medical student education in urban settings.^7^

The COVID-19 pandemic quickly spread to disrupt life around the world, to which medical schools were not immune.^8^ In March 2020, the Association of American Medical Colleges recommended medical schools to pull medical students from clinical spaces. For pre-clinical students, disruption was minimized, as many medical schools already use asynchronous and online education modalities to deliver their pre-clinical curriculum.^9^ Although medical schools were able to adapt most components of their curricula to an online format, critical in-person clinical experiences of the pre-clinical curriculum, such as preceptorships, were not readily adapted, leading pre-clinical students to feel more unprepared for clerkships.^10^

Transitioning in-person extracurricular activities, such as clinical shadowing, had not been widely explored. Several virtual curricula implemented during the pandemic focused on online didactics or case-based virtual components. One program allowed pre-clinical students to shadow physicians in primary care to surgical specialties, however, little has been written about implementation in emergency department (ED) settings.^11^

The Healthcare Leadership and Management (HLM) Scholars Program is an 8-week long summer program designed for a limited number of Weill Cornell Medicine medical students in between their 1st and 2nd years by the department of emergency medicine at New York-Presbyterian Hospital/Weill Cornell Medicine.^12^ Traditionally conducted in boardrooms, offices, and hospitals in New York, NY, this program—inclusive of journal clubs, executive meetings, and group projects—was seamlessly transitioned to a virtual environment for the student cohort in summer 2020. An important feature of the HLM curriculum, which presented more challenging to adapt to a virtual learning experience, was clinical shadowing of ED patient care and of telemedicine patient visits.

In place of shadowing precepting emergency medicine (EM) attending physicians in both patient care environments, students used videoconferencing software to remotely observe direct patient care during ED clinical shifts, as well as to observe telemedicine patient visits. In this article, we seek to describe the lessons learned in implementing this virtual clinical shadowing experience.

## Materials and Methods

**FIG. 1.**
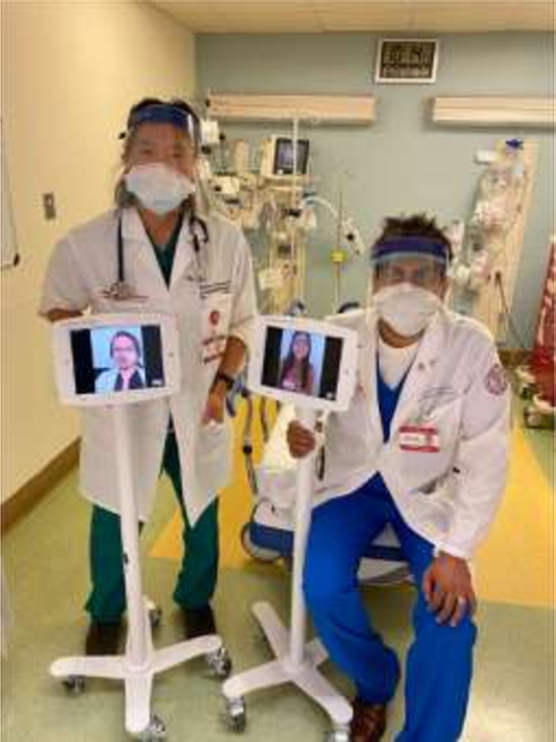
Pre-clinical students virtually shadowing attending physician preceptors.

HLM Scholars telecommuted to virtually shadow EM attending physicians during their ED clinical shifts, as well as on telemedicine patient visits. For ED shadowing, HLM Scholars dialed into a Health Insurance Portability and Accountability Act (HIPAA) compliant Apple iPad on wheels through virtual conferencing by using Zoom.^13^ Precepting attending physicians were responsible for physically rolling around and angling viewing perspective of iPads (Fig. 1). For telemedicine patient visits, Zoom was used to connect three parties: the shadowing HLM Scholar, the precepting attending physician, and the patient receiving care. All HIPAA precautions were taken while using iPads and Zoom.^14^ For each patient encounter, verbal consent for having the medical student join the interaction was obtained by the attending physician preceptor.

A presurvey (Supplementary Data S1) and postsurvey (Supplementary Data S2) were developed to gather responses from the six medical students enrolled in HLM Scholars Program during 2020's COVID-19 pandemic. Questions in the surveys aimed to evaluate the scholars' attitudes toward virtual clinical shadowing. Students were asked background questions about their prior clinical shadowing experiences. The presurvey queried students' expectations for virtual clinical shadowing on a scale of 0–100 (0—extremely negative experience, 50—neutral experience, 100—extremely positive experience) in four learning domains.

The coding schema for domains was created by two investigators (R.T., M.M.) and reviewed by the remainder of the research team. The investigators developed codes *a priori* based on the context of the study and final survey design, with initial revisions made during discussions among the research team. Additional revisions to domains and development of the themes occurred during the coding process according to what emerged from the data *de novo*. Discrepancies in coding were discussed and resolved by investigator consensus so that in the end all coding was reviewed and confirmed by the study team. The presurvey asked students to voice the anticipated benefits and drawbacks of virtual shadowing in a free response format.

Scholars completed a postsurvey with the same questions after completing virtual clinical shadowing to determine whether there was any shift in attitudes and perceptions during the 8-week program.

The surveys were distributed through email and submitted through Qualtrics (Qualtrics, Provo, UT). Consent was implied by completing the survey. No identifiable personal information was collected to ensure anonymity between students. Descriptive statistics were used for analysis. Responses to open questions were analyzed qualitatively using thematic analysis performed by authors.

This study was reviewed and determined to be exempt by the Weill Cornell Medicine Institutional Review Board.

## Results

During the 8-week summer program, all six pre-clinical medical students completing HLM Scholars Program telecommuted from three different time zones and six different cities across the United States. The presurvey was completed by all six students; one student did not complete the postsurvey.

The students reported a cumulative of 440 h of in-person shadowing experience before HLM Scholars Program (median 75 h), which included hours performed before medical school and outside our institution. They participated in 77 h of virtual clinical shadowing during the program. Of the 77 h of virtual clinical shadowing, 38 h were for shadowing telemedicine visits (median 9 h, range 6–14, interquartile range [IQR]: 6–12.5) and 39 h (median 9 h, range 3–12, IQR: 6–9) were for shadowing ED patient care.

**FIG. 2.**
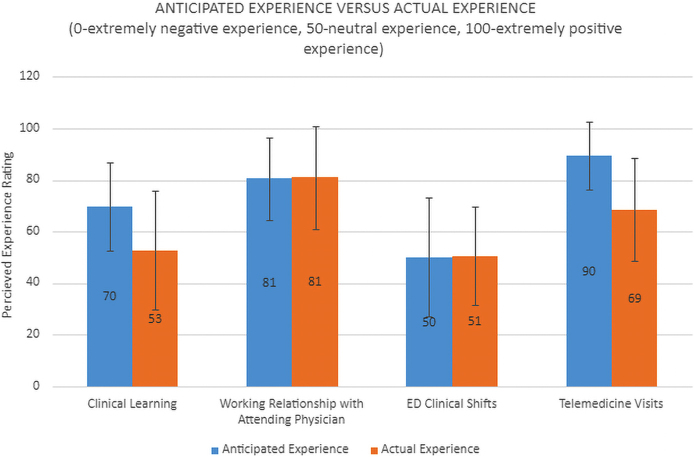
Actual experience rating (compared with anticipated) over four domains: clinical learning, working relationship with attending physician, ED experience, and telemedicine experience.

Figure 2 shows the actual experience rating (compared with anticipated) over four domains: clinical learning, working relationship with attending physician, ED experience, and telemedicine experience. Three main themes emerged from the qualitative data collected by the surveys: the working relationship between the physician and student, logistical considerations, and the holistic shadowing experience (Table 1).

**Table 1.** Summary of Free Text Responses in Four Domains of Virtual Shadowing

Domains	Anticipated (presurvey)	Experienced (postsurvey)
Clinical learning	• Limited interactions with patients and opportunities for direct clinical learning. • Continuous clinical exposure to patients possible	• Provided clinical exposure with simultaneous access to learning tools, e.g., “Ability to independently look up information related to patients' histories and diagnoses to allow for a more intelligent conversation with the attending physicians… [and the] ability to effectively use downtime for other tasks during slow shifts”
Attending relationship	• Limited interactions with attendings, e.g., “lack of personal connection” and “limited teaching experiences”	• More time efficient, e.g., “greater time to chat with the physician in depth,” and “interacted with multiple different attendings”
ED experience	• Limited observation of the clinical setting, e.g., “harder to fully experience and understand operational environment of clinical setting”	• Limited observation of the clinical setting, e.g., “more difficulty seeing the ‘bigger picture’ of what's going on in the department.” • Limitations of, e.g., “not being able to interact directly with patients.” and “not being able to see procedures clearly” • Logistical benefits, e.g., “social distancing” and “easy to fit into schedule…flexible timing”
Telemedicine experience	• Provided exposure to telemedicine, e.g., “gaining insight into telemedicine/logistics of telehealth.” • Expected technical difficulties	• Exposure to telemedicine setting, e.g., “saw behind the scenes of telemedicine visits.” • Technical difficulties, e.g., “not being able to see [telemedicine functions] clearly,” “often difficult to hear,” “connection issues at times”

ED, emergency department.

## Discussion

Across the four domains, three main themes emerged from the qualitative data collected by the surveys: logistical considerations, the physician–student working relationship, and the holistic shadowing experience. These themes were supported by the quantitative survey responses and through anecdotal discussions with participating physicians and HLM Scholars.

Virtual clinical shadowing had several logistical benefits. It was easy to implement, as it leveraged technology already established. For telemedicine visits, physicians and students had been using Zoom for videoconferencing for several months leading up to the program. For ED clinical shifts, the physicians were comfortable using the portable iPads, as they frequently use them to call translator services. Students learned a novel skillset. Before any virtual shadowing, our students watched a 1-h asynchronous training through our Center for Virtual Care, which prepares telemedicine clinical providers on best practices including camera position, lighting, and nonverbal communication.

We recommend brief training to prepare students for a virtual shadowing experience, as students anecdotally commented that this training was insightful and worthwhile. Shadowing virtually reduces travel time and scheduling conflicts for pre-clinical students. Students commented on the overall efficiency of their workday, as they could hop between project team meetings and shadowing shifts without the time delays of wardrobe changes or transit times—a potential postpandemic educational solution for brief preceptor shadowing with long transit times.

Several technical logistic challenges presented themselves, as well. Both physicians in the hospital and students participating remotely reported occasional connection instability. This is especially important to consider for students from underprivileged backgrounds, including students in rural areas or students in developing countries, who may not have access to the devices or infrastructure required to effectively participate in remote learning.^15,16^ For ED clinical shifts, difficulty hearing conversations and observing procedures were common complaints due to intermittent poor audio and video quality, both compounding to make it more difficult to connect with patients as compared with in-person interactions.

Virtual clinical shadowing platform enhanced the physician–student relationship. Informal debriefings revealed that physician preceptors felt more responsible for their students and, thus, were likely more engaged in the mentoring experience. Physicians had to remain cognizant of their students' visual perspective, including height and direction of gaze, to ensure that students could participate effectively. Physicians needed to purposefully introduce students to patients and other health care team members.

From the student's perspective, the virtual shadowing experience met its relatively high expectations (anticipated 81 vs. actual 81). Students enjoyed having real-time access to references and databases, allowing them to look up relevant clinical information in parallel with a patient visit. Conversely, students shadowing clinical care in-person would wait until after a patient visit to access similar reference material, which may delay students' contextualization of physician–patient conversations. All in all, by researching in parallel while virtually shadowing patient care, students were prepared to engage in higher level conversations with their physician preceptor, leading to more fruitful physician–student relationships and learning environments.

Virtual clinical shadowing was not a true replacement for future physicians seeking a holistic education.

First off objective survey responses suggest clinical learning was suboptimal, not meeting students' anticipated expectations (anticipated 70 vs. actual 53). Although an individual patient visit or teaching conversation may be reasonably performed through iPad, postsurvey student commentary suggests that several educational opportunities that exist in-person are lost. In particular, students articulated missing out on physically interacting with patients and not being able to see procedures clearly—two crucial components of clinical shadowing.

Second, though modest expectations were met within the ED patient care (anticipated 50 vs. actual 51), many shortcomings were observed with virtual shadowing in this domain. Students explicitly disliked the lack of peripheral sensory signals—for example, their inability to experience the “bigger picture” from an iPad in a dynamic environment such as the ED. Physician preceptors commented how students were inherently less integrated into the care team and could not proactively observe without the abilities to move throughout the physical space or to rotate their line of sight.

Lastly, even telemedicine patient visits showed room for improvement from an educational experience. Students' objective survey responses demonstrated that their experience of virtual shadowing, although above average, fell markedly short of expectations (anticipated 90 vs. actual 69). Students expressed their desire to see more “behind the scenes” of a telemedicine visit—for example, how physicians stage their own physical space, organize desktop windows, or click through a patient's chart—all of which are challenging to observe from a Zoom window.

Of note, in prior years, students shadowed telemedicine physician preceptors in-person. So, despite the clinical care being provided over telemedicine, unique challenges likely prevented an ideal student learning experience. If these unique challenges can be overcome, telemedicine visits may be a more appropriate setting for student learners to virtually shadow, more so than ED clinical shifts, since patients are seen in series (rather than in parallel), in brief episodes of care (rather than potentially prolonged ED stays), and interactions are contained to a two-dimensional screen (rather than in a dynamic three-dimensional space). Virtual shadowing may provide a long-term opportunity to educate medical students in telemedicine, especially as the pandemic has solidified telemedicine's place in health care.

Several limitations are acknowledged in this study. Our sample size was limited to the six medical students accepted into the HLM Scholars Program during the COVID-19 pandemic. In addition, the presurvey and postsurvey responses were not matched. Given the nature of medical school course evaluation, we believed anonymous survey responses were important to elicit genuine responses from students. Lastly, the presurvey data have six responses, whereas the postsurvey data have five responses, as one student respondent was lost to follow-up. Owing to these factors, we are unable to draw any statistically significant conclusions from the quantitative survey data.

## Conclusion

Our HLM Scholars Program established a virtual clinical shadowing program, which allowed preclinical students to shadow both ED clinical shifts and telemedicine visits remotely. Although counterintuitive, the virtual platform enhanced the physician–student relationship. Implementation was seamless given the existing use of iPads by physicians for translator services and Zoom by medical students in medical education, although it is important to remain wary of technical difficulties. We contend that virtual clinical shadowing is a viable model for providing pre-clinical students with clinical experience when in-person ED clinical shift shadowing is not possible; however, it should not be considered a full replacement for real in-person clinical experience. Instead, the implementation of a virtual clinical shadowing learning experience may be better suited to telemedicine visits.

## Supplementary Material

Supplemental data

Supplemental data

## Abbreviations Used

ED: emergency department
EM: emergency medicine
HLM: Healthcare Leadership and Management
IQR: interquartile range
