# Supplementary material for: Virtual Clinical Shadowing for Pre-Clinical Medical Students in an Emergency Medicine-Based Leadership Course
Source: Telemed Rep. 2021 Oct 27;2(1):233–8. doi: 10.1089/tmr.2021.0019 (PMC9049813; doi:10.1089/tmr.2021.0019)
Supplement: Supplemental data [file Supp_Data1.pdf]

Virtual Clinical Shadowing Post-Survey - Healthcare Leadership and Management Scholars

Q1 I virtually shadowed in-person patient care for a total of ___ hours:

	0 10 20 30 40 50 60 70 80 90 100
Number of Hours ()	

Q2 I virtually shadowed Telemedicine visits for a total of ___ hours:

	0 10 20 30 40 50 60 70 80 90 100
Number of Hours ()	

Q3 My experience of Virtual Clinical Shadowing, in respect to the following aspects was
(0 - extremely negative experience, 50-neutral experience, 100 - extremely positive experience):

	0 10 20 30 40 50 60 70 80 90 100
Clinical Learning ()	
Working relationship with Attending Physician ()	
Understanding operational environment of Emergency Department ()	
Understanding logistical requirements of conducting a Telemedicine visit ()	

Q4 Some benefits of Virtual Clinical Shadowing were:

____

Q5 Some drawbacks of Virtual Clinical Shadowing were:

____

Q6 My experience with Virtual Clinical Shadowing of Clinical Shifts was:

- ○ A much better experience than in-person clinical shadowing (1)
- ○ A slightly better experience than in-person clinical shadowing (2)
- ○ Neither better or worse than in-person clinical shadowing (3)
- ○ A slightly worse experience than in-person clinical shadowing (4)
- ○ A much worse experience than in-person clinical shadowing (5)

Q7 My experience with Virtual Clinical Shadowing of Telemedicine Shifts was:

- ○ A much better experience than in-person Telemedicine shadowing (1)
- ○ A slightly better experience than in-person Telemedicine shadowing (2)
- ○ Neither better or worse than in-person Telemedicine shadowing (3)
- ○ A slightly worse experience than in-person Telemedicine shadowing (4)
- ○ A much worse experience than in-person Telemedicine shadowing (5)
