# Supplementary material for: Virtual Clinical Shadowing for Pre-Clinical Medical Students in an Emergency Medicine-Based Leadership Course
Source: Telemed Rep. 2021 Oct 27;2(1):233–8. doi: 10.1089/tmr.2021.0019 (PMC9049813; doi:10.1089/tmr.2021.0019)
Supplement: Supplemental data [file Supp_Data2.pdf]

Virtual Clinical Shadowing Pre-Survey - Healthcare Leadership and Management Scholars

Q1 In the past, I have clinically shadowed in-person patient care for a total of ___ hours:

	0 10 20 30 40 50 60 70 80 90 100
Number of Hours ()	

Q2 In the past, I have shadowed Telemedicine visits for a total of ___ hours:

	0 10 20 30 40 50 60 70 80 90 100
Number of Hours ()	

Q3 I anticipate my experience of Virtual Clinical Shadowing, in respect to the following aspects to be
(0 - extremely negative experience, 50-neutral experience, 100 - extremely positive experience):

	0 10 20 30 40 50 60 70 80 90 100
Clinical Learning ()	
Working relationship with Attending Physician ()	
Understanding operational environment of Emergency Department ()	
Understanding logistical requirements of conducting a Telemedicine visit ()	

Q4 I anticipate some benefits of Virtual Clinical Shadowing to be:

____

Q5 I anticipate some drawbacks of Virtual Clinical Shadowing to be:

____

Q6 I believe Virtual Clinical Shadowing of Clinical Shifts will be:

- ○ A much better experience than in-person clinical shadowing (1)
- ○ A slightly better experience than in-person clinical shadowing (2)
- ○ Neither better or worse than in-person clinical shadowing (3)
- ○ A slightly worse experience than in-person clinical shadowing (4)
- ○ A much worse experience than in-person clinical shadowing (5)

Q7 I believe Virtual Clinical Shadowing of Telemedicine Shifts will be:

- ○ A much better experience than in-person Telemedicine shadowing (1)
- ○ A slightly better experience than in-person Telemedicine shadowing (2)
- ○ Neither better or worse than in-person Telemedicine shadowing (3)
- ○ A slightly worse experience than in-person Telemedicine shadowing (4)
- ○ A much worse experience than in-person Telemedicine shadowing (5)
